# An open science pathway for drug marketing authorization—Registered drug approval

**DOI:** 10.1371/journal.pmed.1003726

**Published:** 2021-08-09

**Authors:** Florian Naudet, Maximilian Siebert, Rémy Boussageon, Ioana A. Cristea, Erick H. Turner

**Affiliations:** 1 Université de Rennes 1, CHU Rennes, Inserm, CIC 1414 (Centre d’Investigation Clinique de Rennes), Rennes, France; 2 Université Claude Bernard Lyon 1, CNRS, UMR 5558, LBBE, EMET, Lyon, France; 3 Department of Brain and Behavioral Sciences, University of Pavia, Italy; 4 IRCCS Mondino Foundation, Pavia, Italy; 5 Behavioral Health and Neurosciences Division, VA Portland Health Care System, Portland, Oregon, United States of America; 6 Department of Psychiatry, Oregon Health & Science University, Portland, Oregon, United States of America

## Abstract

Florian Naudet and co-authors propose a pathway involving registered criteria for evaluation and approval of new drugs.

Publisher’s note: This Perspective is one of the two winning Essays of the “Reimagine biomedical research for a healthier future” Essay challenge, launched by the Health Research Alliance in partnership with PLOS. This publication is coordinated with that of the other winning Essay in PLOS Biology. The competition was intended to spark a discussion around the future of biomedical research; publication does not imply endorsement from HRA or PLOS.

## Background

Before drug approval, health authorities like the European Medicines Agency (EMA) and the United States Food and Drug Administration (FDA) evaluate findings from the relevant clinical trials to assess the balance between clinical benefit and safety. When requesting marketing authorization for their drug products, pharmaceutical companies are allowed to choose the indication, design the trials, and choose assessments. In the US, pharmaceutical companies and drug manufacturers must submit full trial protocols to the FDA before those trials can begin. In Europe, companies can, at their discretion, obtain prior scientific advice from the EMA. This consultative process between sponsor and regulator is not fit for purpose, as there is, in practice, no clear a priori consensus on the exact criteria that will be applied to adjudicate success.

Although the FDA lays out a set of a priori rules, all too often, it later bends those rules post hoc. For instance, for esketamine, for treatment of resistant depression, the FDA decided post hoc that a maintenance trial could substitute for a second positive short-term trial [1]. Other examples include nalmefene for alcohol use disorder (approved by the EMA), which was based on a post hoc subgroup analysis of the pivotal trials [2], or eteplirsen for muscular dystrophy (approved by the FDA) despite a lack of clinical evidence [3].

Even the initial standards agreed upon between the sponsor and regulator can be too lax. Too often, trials ask the wrong question: Trials may explore superiority over an inappropriately weak comparator such as placebo when superiority versus an already approved active comparator would be more clinically relevant [4]. Trials can also be underpowered [4], focus on surrogate markers, or omit clinically relevant outcomes [5]. Moreover, the regulator is laissez-faire with respect to trial publication in journal articles, allowing the sponsor to freely choose which findings to include and how to frame them, often diverging starkly from the regulator’s reviews. With few stakeholders aware of these reviews, the journal publication, often rife with selective reporting and spin, becomes the most influential source of information. Consequently, drugs approvals are frequently marred by inaccuracies and contradictions.

Systematic investigations demonstrate that approvals based on weak and limited evidence are the rule rather than the exception [4,5], although there are notable instances where approval was based on strong evidence, such as the recent case of Coronavirus Disease 2019 (COVID-19) vaccines. As a result, more drugs with little, if any, added benefit are brought to the market in a process increasingly reliant on disputable evidence [6] and divorced from public interest.

Some regulators, like the EMA, do not attempt to replicate the sponsor’s analysis. Even the FDA, which reanalyzes individual patient data from the sponsor, does not make the data accessible to independent researchers. The combination of controversial approvals and lack of transparency nurtures justified criticism and decreases societal trust in medicine.

## An open science pathway for drug marketing authorization

We propose to adapt the concept of “registered reports” to the process of regulatory drug approval and marketing authorization. It may provide an innovative, unambiguous, transparent, and trustworthy research pathway.

**Fig 1:**
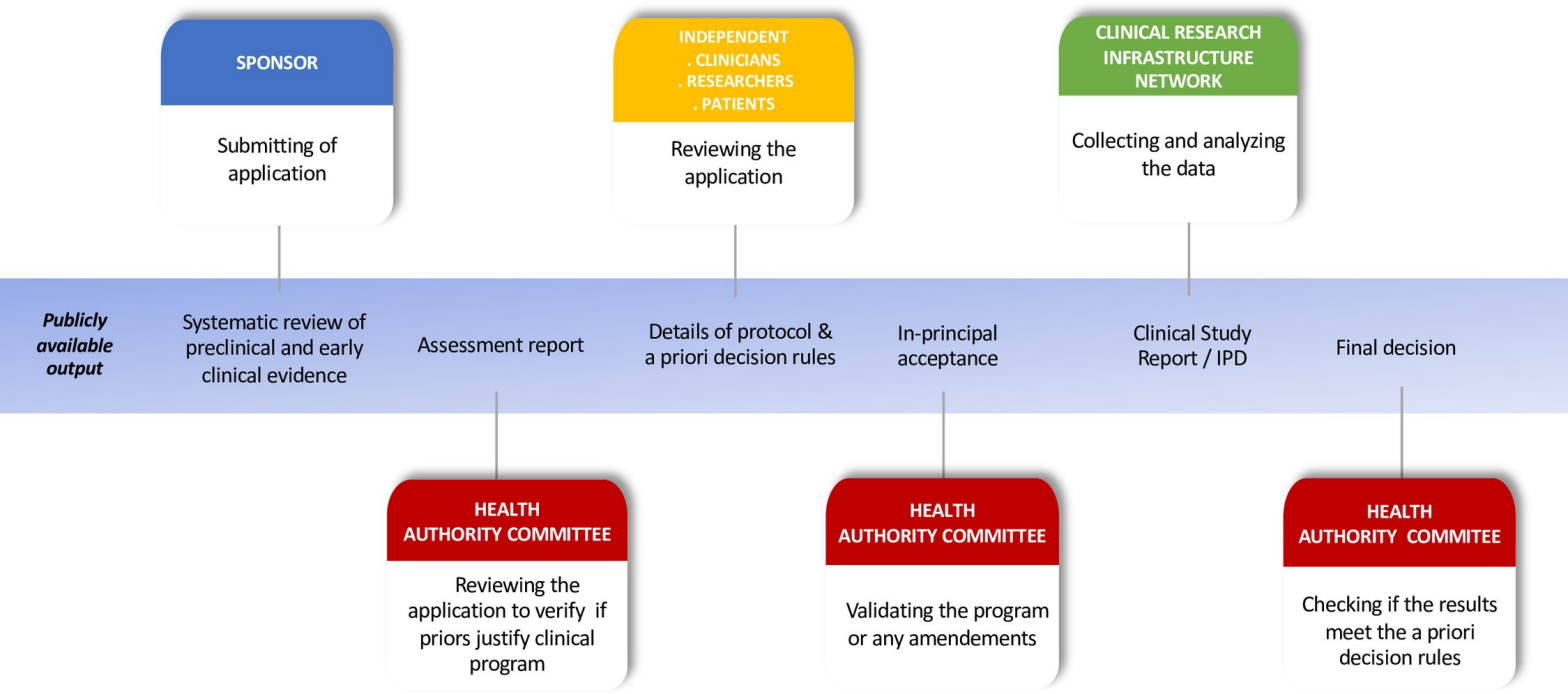
Overview of the registered drug approval pathway. IPD, individual patient data.

Registered reports represent a publishing format premised on “the importance of the research question and on the quality of methodology by conducting peer review prior to data collection” [7]. Transposed into the field of regulatory science, in a registered approval (**Fig 1**), health authorities would be required, a priori, to pose research questions that matter (in terms of patients, interventions, comparator, outcome, and study design) and define adequate criteria for success, with no possibility of bending the rules after data collection.

Any sponsor could propose a registered drug approval program contingent on the presentation of preclinical and early clinical phase evidence for the usefulness of the drug in the context of its research question. Development and peer review of the proposed research program would involve a dedicated committee assembled by the health authority involving multiple stakeholders, independent from the sponsor (e.g., clinicians, researchers, and patients). The FDA already has such advisory committees, but their meetings are scheduled close to the end of the approval process, after trial results are known, placing them at risk for spin and post hoc rule bending. In contrast, in the registered approval scheme, stakeholders will preemptively be involved in the process to provide insights into the value of the research question, as well as on the clinical relevance of the proposed intervention. Insights on appropriate comparators can be provided by living systematic reviews and meta-analyses. Important examples established during the COVID-19 pandemic [8] represent a blueprint for delineating the future agenda for evidence generation. Comparative effectiveness will be systematically considered, as will the use of core outcome sets (i.e., an agreed-upon standardized list of outcomes to be measured and reported in all trials for a specific clinical area [9]). The required number of direct pivotal trials as well as study designs will be set a priori for the research program, including large simple trials and nonambiguous criteria for success, e.g., 2 positive confirmatory studies with low risk of bias and a prospective meta-analysis. These criteria will not only define the prespecified analyses and criteria for statistical significance, but also the precise criteria for clinical relevance, i.e., a minimal clinically important difference defined on clinically relevant outcomes or net benefit. Health authorities will ensure a thorough peer review process of the protocols.

Following a positive outcome of the peer review process, drugs would be provisionally granted approvals for specific use in the clinical trials of the registered drug approval research program. In case of any deviations from the protocol, the committee in charge of the registered approval would agree on the best way to handle them before unblinding and statistical analysis. Subsequently, an approval would be granted for clinical use and marketing authorization provided that (1) the research program adhered to the registered methodology; and (2) predefined criteria for success were met. Approval would require that both conditions have been met.

Transparency would be paramount, with sharing of protocols, followed by aggregated data and individual patient data. Transparency would be guaranteed, regardless of whether the drug is approved or not. Clinical trial registries, such as ClinicalTrials.gov, could evolve to support the uploading of all these documents. Prospective registration on these public registries is the norm for clinical trials. Moreover, they have evolved to also include trial results, and, thus, are in a privileged position to expand toward incorporating more comprehensive open science tools empowering Findability, Accessibility, Interoperability, and Reusability (FAIR) access to any study related data. Undoubtedly, such a radical push toward transparency in planning, conducting, and reporting research, if promoted by an influential national or transnational regulatory authority, would have profound consequences for the entire field of biomedicine. Last, all output of the research program would “feed” the living meta-analysis without delay so as to inform future registered drug approvals and to ensure the integrity of the entire scientific process from planning to publication of the results and data.

## Implementation challenges

Practical implementation and acceptability of this pathway could be challenging. Owing to the complexity and resources needed for registered drug approvals, a centralized approach would be desirable. This approach requires the endorsement and harmonization of the pathway among the various health authorities, who currently follow distinct procedures. Nevertheless, the EMA and FDA have already initiated collaboration protocols on drugs [10]. Joining forces on an initiative that fosters sound science and scientific integrity seems a compelling reason to strengthen such collaborations.

The most obvious obstacle to this proposal is sponsor buy-in. Adopting such a pathway would require major structural changes in drug laws, which would almost certainly be met by heavily financed opposition and lobbying by drug companies. And because much agency funding comes from drug company user fees, sponsors may be reluctant to lose control over the process by which trial results, which they have long regarded as “trade secrets,” are disseminated. Other obstacles could include differences in ethics criteria and specialty-specific clinical guidelines across countries. Therefore, a first implementation initiative would be aimed at encouraging sponsor participation. We propose the pathway as optional for selected drugs that may be eligible, akin to a “golden” approval pathway that would be accompanied by additional and specific incentives.

One such incentive for sponsors could be that, through a single application, this process simplifies the process of access to all markets, owing to the potential involvement of an international agency. A more important incentive is that approval via this pathway honors the ethical duty of all stakeholders toward trial participants who altruistically put themselves at risk and can hence strengthen trust in science. Drugs approved via this pathway would thus gain a seal of quality, affording them a competitive advantage in the marketplace, resulting in a financial incentive for the sponsor.

Nevertheless, it is also important that appropriate incentives be allocated to all involved stakeholders, independently of the results and with a particular focus on data generators.

In such a pathway, one may balance the cost of an independent and robust system of evidence generation with the savings generated by ending the continuous flow of costly drugs, with little added value and concrete risks, approved within the current system.

While these challenges are difficult to overcome, the minimum we believe can and should be achieved is that any trial intended to support drug approval should be submitted as a registered report. Such a publication would not prevent the regulatory agency from post hoc rule bending and approving a drug that shouldn’t have been approved, but, at least, clinicians, patients, and policymakers would be apprised of the true outcomes of all trials. Compared to trials disseminated through conventional publication pathways, stakeholders would likely find such registered report publications more credible and informative.

## Abbreviations

COVID-19: Coronavirus Disease 2019
EMA: European Medicines Agency
FAIR: Findability, Accessibility, Interoperability, and Reusability
FDA: Food and Drug Administration
